# Current insights and prospects for the pathogenesis and treatment of clinical manifestations associated with Down syndrome through neurotransmitter, inflammatory, and oxidative stress pathways

**DOI:** 10.3389/fphar.2025.1592277

**Published:** 2025-04-28

**Authors:** Fawaz Alasmari, Ashfaq Ahmad, Sary Alsanea, Alaa M. Hammad, Walid Al-Qerem

**Affiliations:** ^1^ Department of Pharmacology and Toxicology, College of Pharmacy, King Saud University, Riyadh, Saudi Arabia; ^2^ King Salman Center for Disability Research, Riyadh, Saudi Arabia; ^3^ Department of Pharmacy Practice, College of Pharmacy, University of Hafr Al Batin, Hafr Al Batin, Saudi Arabia; ^4^ Faculty of Pharmacy, Al-Zaytoonah University of Jordan, Amman, Jordan

**Keywords:** Down syndrome, neurobehaviors, neurotransmitters, inflammation, oxidative stress, multi-omics

## Abstract

Individuals with Down syndrome exhibit various changes in the human body systems, including alterations in the ocular, neurological, and dermatological systems. Especially, preclinical and clinical studies have determined Down syndrome patients to possess reduced intellectual and cognition abilities, which neurobehavioral effects are associated with altered molecular markers in the brain. For instance, neuroinflammation and increased brain oxidative stress are reported in animals models of Down syndrome, and the reversal of those markers lead to positive effects. Dopaminergic and serotonergic neurons are altered in individuals with Down syndrome, with dopamine and serotonin secretion reduced and their transporters upregulated. Hence, blocking reuptake of dopamine and serotonin might improve Down syndrome behavioral impairments. Norepinephrine loss was observed in a mouse model of Down syndrome, and treatment with a β2 adrenergic receptor agonist improved behavioral symptoms. Moreover, targeting certain glutamatergic receptors, particularly in the hippocampus, might correct the glutamatergic dysfunction and altered behaviors. Inverse agonists or antagonists of GABAergic receptors suppress GABA’s inhibitory role, an effect associated with improved cognition behaviors in models of Down syndrome. Reports also suggest partial involvement of the histaminergic system in the impairment of memory function observed in Down syndrome. Finally, cholinergic system alteration has been reported, but the therapeutic role of its modulation needs further investigation. This review collects and reports multi-Omics Studies on Down syndrome, the crucial roles of inflammation, oxidative stress independently as well as role of oxidative stress in pregnancies with Down Syndrome and biomarkers of maternal diagnosis of Down syndrome. This review further explained the role of neurotransmitter pathways in Down syndrome pathogenesis, prognosis and therapeutic intervention for Down syndrome and future directions for interventions.

## 1 Introduction

Down syndrome is a genetic disorder caused by an additional chromosome, either a full or partial copy of chromosome 21 (trisomy 21) which results in distinct neurobiological traits, neurobehavioral changes, and clinical features (Antonarakis et al., 2020). Genetic factors are critical determinants of fetus growth and its malformations as well as of the chromosomal abnormality (Antonarakis et al., 2020), which typically occurs during meiosis (Ataman et al., 2012). Individuals with Down syndrome share a number of common characteristics such as craniofacial abnormalities, periodontal disease (Ghaffarpour et al., 2024), learning impairments, and early-life hypotonia (Antonarakis et al., 2004). Some individuals present with variable phenotypes, including leukemia (both acute megakaryoblast leukemia and acute lymphoblastic leukemia), along with atrioventricular septal defects in the heart (Asim et al., 2015). Physical characteristics of individuals with Down syndrome include weak muscular tone, a small chin, slanted eye, a single palm crease, a flat nasal bridge, and a small upper jaw mouth and wide tongue (Sinet et al., 1994). Posterior cortical atrophy was reportedly manifested in a Down syndrome individuals with Alzheimer’s disease (Rodríguez-Baz et al., 2025).

All individuals with Down syndrome have varying degrees of hypotonia and developmental delay. A recent study found that intellectual and developmental disabilities were compromised in individuals with Down syndrome (Carvalho et al., 2025). Ongoing study elaborated that traditional cognitive dual tasks and everyday tasks, such as talking and typing on a cell phone, had a greater impact on individuals with Down syndrome than on the control group. With the rising prevalence of multitasking in daily life, including dual-task activities in rehabilitation programs could help enhance functional mobility in this population. A study conducted between December 2020 and February 2023 on the children (3–17 years of age) having anorectal malformations (ARM) and Hirschsprung’s disease (HD) both with and without Down syndrome reported that Children with ARM and HD with Down syndrome underwent lower score than without Down syndrome on motor development, adaptive skills, feeding, toilet training, sleep and social development (Rajasegaran et al., 2024). Motor development, especially standing position and walking ability, is delayed in children with Down syndrome (Malak et al., 2015). Their poor language and communication abilities are primarily caused by speech delay, poor articulation brought on by their small oral cavity, and a lack of language comprehension (Agarwal Gupta and Kabra, 2014). This study further explained despite such comparative setbacks, these patients are typically cheerful, kind, loving, and outgoing while a few of them enjoy listening to music. The distinctive physical characteristics of Down syndrome allow skilled medical professionals to easily make the clinical diagnosis; hence, patients are typically identified at birth or soon after. However, findings of a prior study explained that certain ethnic groupings, older patients, premature newborns, and mosaicism may present diagnostic challenges (Kazemi et al., 2016); furthermore, every person with Down syndrome has unique medical requirements. Presenting a prenatal diagnosis of Down syndrome should be done in person or over the phone at a predetermined time, with parents reportedly appreciating the compassionate way in which medical doctors can communicate such diagnoses (Skotko et al., 2009). Study under discussion emphasize in connecting parents with nearby Down syndrome support groups and other resources, doctors should accurately inform them about medical issues linked to Down syndrome. Recently reported study explained that awareness of the neurobehavioral changes or neurobiological alterations, such as neuroinflammation or impairment of neurotransmitters, that occur in the brains of Down syndrome individuals is important to avoid misattribution to intellectual disability (Baumer and O’Neill, 2022).

## 2 Multi-omics studies on Down syndrome

As it is evident that Down syndrome is a genetic disorder characterized by presence of an extra chromosome, trisomy 21, single-cell multi-omics map of haematopoietic stem cells human fetal showed that these alterations in chromatin organization leading to change in gene expression dynamics and oxidative stress create an environment that is suitable or favorable to hematological changes like pre-leukemia and post-leukemia (Marderstein et al., 2024). Genetic alterations in Down syndrome have been associated with changes in protein expression, although the underlying mechanisms remain largely unknown. Postmortem brain analyses employing multi-omics approaches have demonstrated that the hippocampus and cortex share transcriptomic signatures at both the gene and transcript levels, as well as proteomic dysregulation (Rastogi, 2021). To gain further insight into the pathophysiology of Down syndrome in humans, studies using mouse models have incorporated additional omics strategies, such as metabolomics, including lipidomics and elementomics (notably metallomics), which are anticipated to contribute significantly to the understanding of its molecular underpinnings. However, no proteins were found to exhibit differential expression in the Ts1Cje mouse brain during neonatal and postnatal stages based on 2D-electrophoresis proteomics (Ishihara et al., 2014). Omics analysis of elementomics, with metallomics showed that disruptions in the balance of intrinsic metals such as zinc, copper, and iron may contribute to cognitive impairments in individuals with Down syndrome, as intersectin 1, located in the trisomic region of Down syndrome, is thought to play a role in iron uptake. In fact, elevated levels of non-protein-bound iron in the serum and the iron-binding protein lactotransferrin in the brain have been observed in individuals with Down syndrome (Manna et al., 2016). Based on the multi-omics study the identification of the noninvasive prenatal testing made a tremendous progress to diagnose the Down syndrome in the uterus, fetal structural abnormalities, genetic diseases, and pregnancy-related diseases before birth thus could offer evidence for intrauterine treatment or selectively termination of pregnancy. Among these biomarkers of abnormalities studied in multi-omics study, cell-free DNA, allows for a definitive diagnosis in early pregnancy for fetal diseases, including Down syndrome and other common aneuploidies (Wang et al., 2022). The association between mitochondrial DNA damage (mitochondrial DNA is different from nuclear DNA as mitochondrial DNA also plays a role in regulating cell metabolism, growth, and apoptosis) and Down syndrome has been reinforced by evidence from brain tissue analysis in individuals with Down syndrome (Coskun and Busciglio, 2012) making it a potential target in this condition (Izzo et al., 2018). Other than mitochondrial DNA damage, some useful pharmacological activators of Peroxisome Proliferator-Activated Receptors γ coactivator 1 alpha pathway, including thiazolidinediones, pioglitazones, and bezafibrates, which selectively stimulate peroxisome proliferator-activated receptors and can be evaluated for restoring mitochondrial functions of the DNA in Down syndrome (Landreth et al., 2008).

While research on Alzheimer’s disease gives us important background information on the function of inflammation in brain disease, the triplication of numerous inflammatory-associated genes in Down syndrome clearly generates a distinct inflammatory milieu deserving of more study (Wilcock, 2012). For example, individuals with Down syndrome exhibit early neuroinflammatory phenotypes that change throughout their lives (Flores-Aguilar et al., 2020). This finding is important for identifying features specific to each stage of the disease and for innovating potential anti-inflammatory compounds to prevent dementia in this population (Flores-Aguilar et al., 2020). Extant data indicates that understanding inflammation in Down syndrome will be crucial for advancing research on the state of the brain molecular mechanisms in neurodegenerative diseases, and the inflammatory pathways typically are involved in such diseases (Wilcock, 2012). Down syndrome is associated with neurodegenerative disease so reported data suggest that individuals with Down syndrome could benefit from regular exercise, physical activity, and controlled antioxidant supplements to enhance cognitive abilities (Muchová et al., 2014). Notably, oxidative stress is established to be involved in the etiology of Down syndrome, despite the remarkable cellular defenses intended to combat its toxicity (Muchová et al., 2014). Individuals with Down syndrome experience systemic oxidative stress as indicated by increased activity of several key antioxidant enzymes, decreased whole-blood glutathione level, and elevated plasma uric acid level. These findings are likely related to redox imbalance and represent an antioxidant compensation (Garlet et al., 2013). This review discusses the involvement of inflammatory and oxidative stress pathways as well as the role of neurotransmitters in the pathophysiology of Down syndrome.

## 3 Role of inflammation in Down syndrome

**FIGURE 1:**
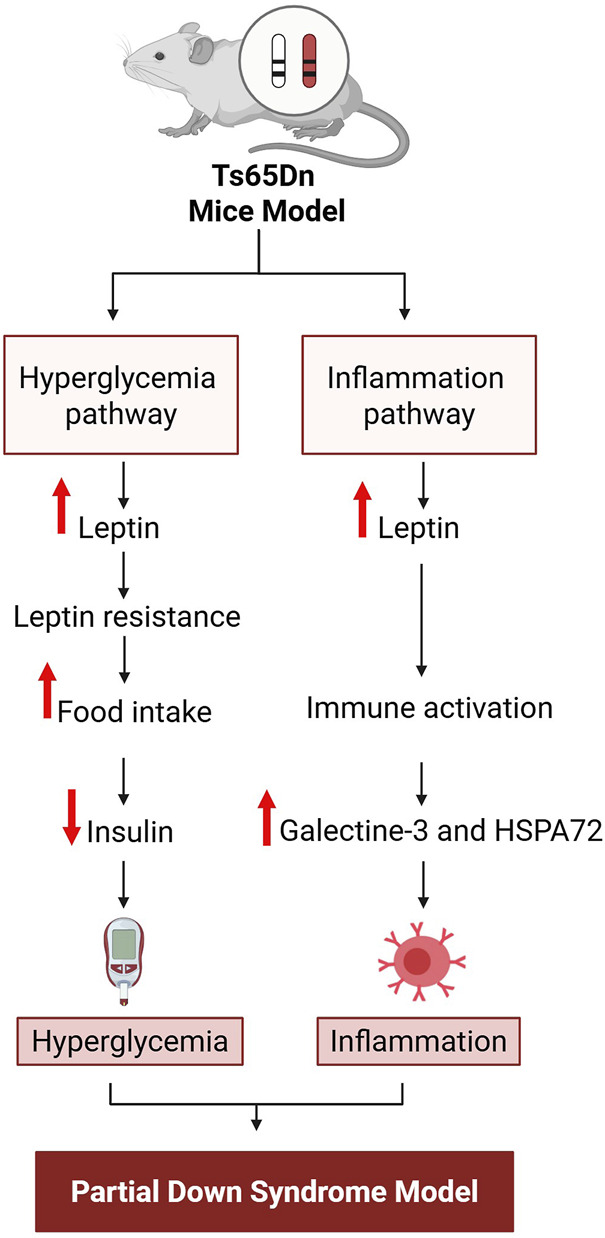
Sequence of events in the impaired metabolic-inflammation axis implicated in Down syndrome. HSPA 72, Heat shock protein A 72. Figure was created with BioRender.com.

A direct association between inflammation and Down syndrome has not been demonstrated, although an indirect relation is supported by interlinking immunity (Ram and Chinen, 2011), metabolic syndrome (Dierssen et al., 2020) and oxidative stress along with Alzheimer’s disease (Hajjo et al., 2022; Ekundayo et al., 2024). A study conducted on Ts65Dn mice, a partial trisomy model of Down syndrome, observed elevation of leptin, galectin-3, and heat shock protein A 72 (HSPA 72) (Fructuoso et al., 2018), which are linked with autoimmunity (de Oliveira et al., 2015). Moreover, increased leptin, glucose, and thyroid hormone imbalance have been reported in Egyptian children with Down syndrome (Yahia et al., 2012). Previous studies on adolescents with Down syndrome measured the hematological, biochemical and cardiometabolic variables including serum levels of complement factor 3 and 4, along with C-reactive protein in the UP&DOWN studies (Castro-Piñero et al., 2014). The idea that Down syndrome involves impairment of metabolic-inflammation axes is widely accepted and seems logical. The supporting findings for this concept are illustrated in Figure 1.

In Ts65Dn mice, two pathways, metabolic-inflammation pathways, have been identified (Fructuoso et al., 2018). The first involves hyperglycemia; this begins with increased food intake due to increased leptin resistance due to increase leptin levels, which in turn block insulin secretion, leading to the onset of hyperglycemia. The second is an indirect pathway for activation of inflammation that also originates with increased leptin, as leptin causes immune system activation by increasing the levels of galectin-3 and HSPA 72, which in turn activate inflammation. These conceptual pathways are based on previously reported data (Fructuoso et al., 2018).

Involvement of the immune system is also supported by human data. Compared to euploid individuals, young people with Down syndrome exhibit higher levels of cytokines, chemokines (Veloso et al., 2025), and macrophage inflammatory protein-beta (Flores-Aguilar et al., 2020). Additionally, conditioned media of mixed cortical primary cultures from second trimester fetuses with Down syndrome feature was associated with increased cytokine expression (Flores-Aguilar et al., 2020). However, children with Down syndrome exhibit a significant drop in serum level of all examined cytokines, with clear distinctions between male and female children (Tarani et al., 2020) while this study also examined how neurotrophins and immune system pathways are disrupted in prepubertal children with Down syndrome (Tarani et al., 2020), while young adults with Down syndrome exhibit increased numbers of rod-like microglia (Flores-Aguilar et al., 2020). In the Ts65Dn mouse model, neuroinflammation can be inhibited by a moderate dose of resolvin E1, with 4 weeks of administration resulting in a significant decrease in memory loss, lower serum levels of pro-inflammatory cytokines, and decreased hippocampal microglial activation (Hamlett et al., 2020). Additionally, chronic consumption of extra virgin olive oil in the same mouse model has also been discovered to improve neuroinflammation, synapse function deficiencies, and memory dysfunction (Li et al., 2024).

Among individuals with Down syndrome who are 50 years of age or older, neuroinflammatory processes have been reported in many brain regions, which could be linked to the early aging process of β-amyloid plaque formation (de Oliveira et al., 2023); postmortem studies on both Down syndrome and Alzheimer’s patients have identified accumulation of extracellular amyloid-β (Aβ) protein plaques in brain and blood vessels (Hartley et al., 2015). Crucially, however, compared to people with sporadic Alzheimer’s disease, those with Down syndrome exhibit a different neuroinflammatory profile (Wilcock et al., 2015). Reported data demonstrate that T cell activation can be managed not only with broad immunosuppressants like Jak inhibitors, but also through a more targeted approach using IL-6 inhibition in Down syndrome (Malle et al., 2023). Interestingly, the protein products of chromosome 21 genes that result in neuroinflammatory reactions observed during brain development of fetuses with Down syndrome can potentially hasten the neuropathogenesis of Alzheimer’s (Wilcock and Griffin, 2013). Likewise, a newly-published study that links inflammatory processes with neuron-derived exosome release reported that exosomal Alzheimer’s disease biomarkers may be impacted by the increased neuroinflammatory state observed in individuals with Down syndrome (Hamlett et al., 2018). These findings suggest a critical role of neuroinflammation in the cognitive behavior deterioration that is co-morbid in both Alzheimer’s disease and Down syndrome.

## 4 Role of oxidative stress in Down syndrome

Oxidative stress is caused by an imbalance between pro-oxidants and antioxidant enzymes (Ahmad et al., 2016) and leads to many diseases, including Alzheimer’s disease (Butterfield and Halliwell, 2019). Studies have shown commonalities between Alzheimer’s disease and Down syndrome, such as presence of Aβ protein in the brain and blood vessels (Hartley et al., 2015). Moreover, Aβ accumulation was observed in Down syndrome (Wiseman et al., 2018). In addition, chromosome 21 anomalies that affect Cu/Zn-superoxide dismutase and BACH1 (a transcriptional inhibitor of hemoglobin oxygenase 1 [HO-1]), are implicated in both diseases (Halliwell and Gutteridge, 2015). Interference with the function of HO-1, a crucial cellular antioxidant that breaks down the pro-oxidant free heme (Halliwell and Gutteridge, 2015) can lead to oxidative stress (Perluigi et al., 2014; Di Domenico et al., 2015). Other proteins encoded on chromosome 21 have been implicated in the formation of hyperphosphorylated Tau tangles (Di Domenico et al., 2018) and Aβ plaques, creating a cycle of neural damage in Down syndrome. Notably, a review highlighted that t has been proposed that individuals with Down syndrome experience abnormal oxidative stress, potentially due to increased activity of Cu/Zn superoxide dismutase (SOD1) (Gardiner, 2010); which causes an excess of the reactive oxygen species (ROS) and hydrogen peroxide (H_2_O_2_) an important precursor of hydroxyl radical (Campos and Casado, 2015). When the production of reactive oxygen and nitrogen species (RONS) surpasses the capacity of antioxidant defense systems to eliminate them, an imbalance between RONS generation and antioxidant protection can lead to oxidative/nitrosative damage to cellular components (DNA, proteins, lipids, and sugars), a condition known as oxidative/nitrosative stress which is also suggests oxidative stress as a potential significant factor in the pathophysiology of Down syndrome (Roizen and Patterson, 2003; Campos and Casado, 2015).

### 4.1 Role of oxidative stress in pregnancies and Down syndrome

**FIGURE 2:**
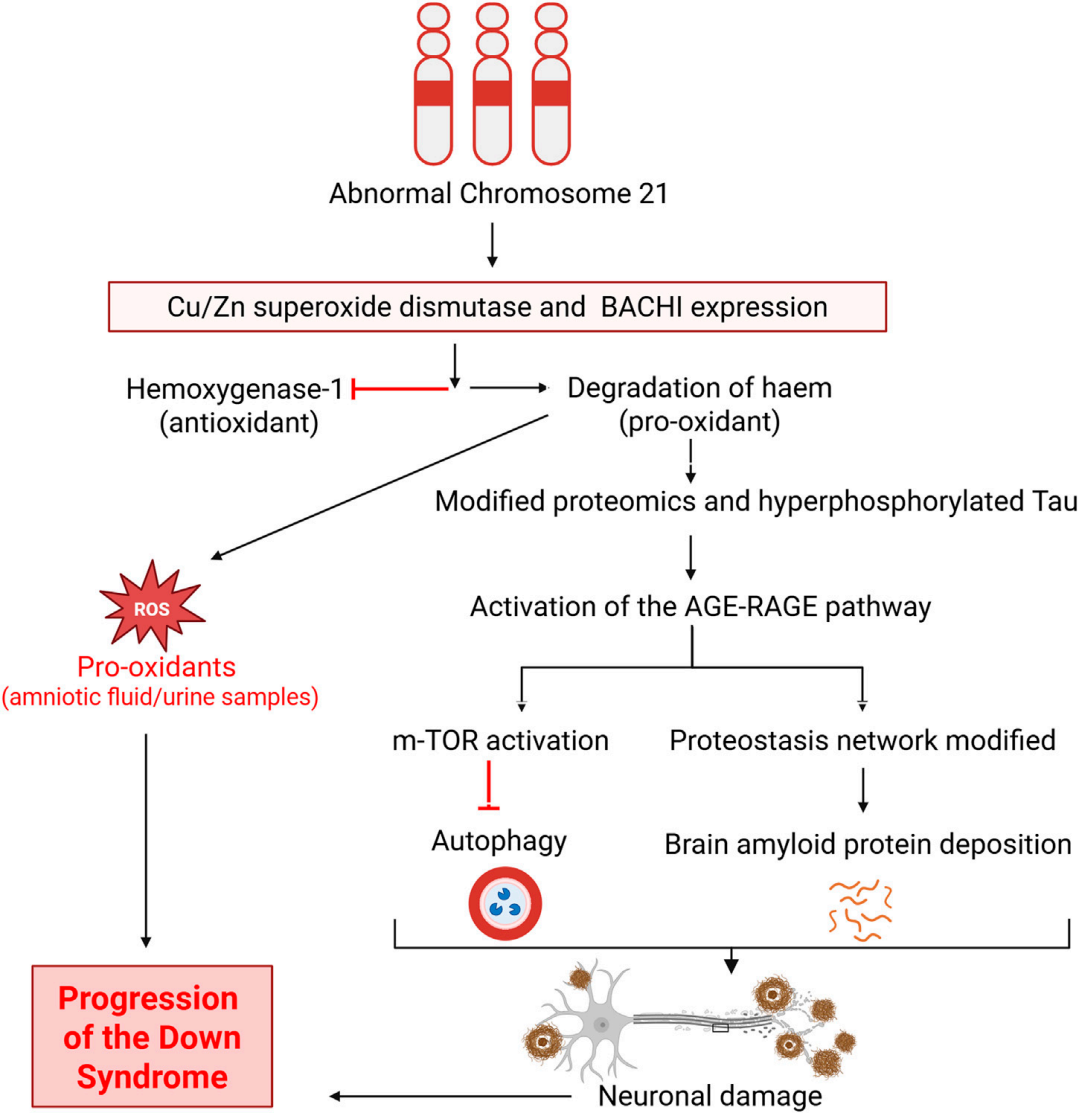
Involvement of oxidative stress in the pathogenesis and progression of Down syndrome. BACH1, Transcription factor BTB domain and CNC homology 1; mTOR, Mammalian target of rapamycin; AGE-RAGE, Advanced glycation end product-Receptors of advanced glycation end product. Figure was created with BioRender.com.

Observations of oxidative damage in aborted fetuses with Down syndrome (Busciglio and Yankner, 1995) and in amniotic fluid obtained from mothers carrying a Down syndrome fetus (Perluigi et al., 2011) strengthen the idea that oxidative stress is involved in the manifestation of Down syndrome. Earlier study reported that not only depression, anxiety and anger but also posttraumatic stress in the mother will change the neurobiology of prenate (Thomson, 2007). Evidence of oxidative DNA damage has also been demonstrated in the form of increased lipid peroxidation and isoprostane 8,12-iso-iPF2α-VI in urine from children and amniotic fluid from fetuses with Down syndrome (Pallardó et al., 2006; Perrone et al., 2007). However, another investigation found the elevations of these lipid peroxidation markers in the blood of live individuals with Down syndrome to not be associated with either SOD1 or glutathione peroxidase (Strydom et al., 2009). Nonetheless, proteomic analysis has also confirmed oxidative damage to diverse proteins in amniotic fluid, such as transferrin, ceruloplasmin, apolipoprotein A-I, retinol-binding protein 4, collagen α1(V), and complement C9 (Perluigi et al., 2011). Hence, in the light of above-mentioned literature, it is strongly suggested that oxidative damage is involved in the manifestation and progression of Down syndrome. Maternal oxidative stress during the pregnancy and its impact on the alteration of neurobiology can be assessed by using markers of oxidative stress process which is ischemia-modified albumin (IMA). Albumin produced during ischemia is often a result of oxidative stress, and the measurement of serum IMA levels has been introduced as a biomarker for several pregnancy-related conditions, including asphyxia (Dursun et al., 2012), acute coronary syndrome (Chawla et al., 2006) and diabetic ketoacidosis in children (Ayar et al., 2019). Serum measurement of IMA during pregnancy can predict maternal oxidative stress which can damage different proteins and ultimately development of Down syndrome. One of the oxidative stress protein deficiencies like alpha-1-antitrypsin has been reported to involved in the pathogenesis of Down syndrome half of century ago (Arnaud et al., 1976). These oxidatively damaged and modified proteins have been implicated in modified glucose metabolism, mammalian target of rapamycin (mTOR) signaling, insulin resistance leading to proteostasis (Di Domenico et al., 2015; Barone et al., 2017; Di Domenico et al., 2018), and impaired brain function due to neuronal damage. Notably, activation of mTOR signaling and consequent inhibition of autophagy leads to the deposition of non-degrading moieties like amyloid protein, which further increases oxidative stress in cells through activation of advance glycation and receptors of advance glycation end products (AGE-RAGE), which in turn leads to further induction of mTOR signaling. Microtubule instability ensues, which impacts mitochondrial trafficking and ultimately causes presynaptic energy shortages. The subsequent loss of ion gradients in presynaptic membranes results in deteriorative Ca^2+^-related changes, which are secondary to the loss of mitochondrial anterograde transport down axons (Reed et al., 2009; Di Domenico et al., 2017; Di Domenico et al., 2018). A visual summary of these adverse events related to oxidative damage is given in Figure 2.

As illustrated in Figure 2, abnormalities in chromosome 21 ultimately lead to the inhibition of antioxidants and increased levels of pro-oxidants. The resultant modified proteome and production of hyperphosphorylated Tau in turn activates the oxidative stress pathway ladder involving AGE-RAGE, which activates m-TOR and the proteostasis network. Both of those then inhibit autophagy and allow deposition of amyloid protein, leading to neuronal damage and progression, culminating in the manifestation of Down syndrome. Deposition of amyloid protein is observed in Down syndrome individuals in a manner similar to Alzheimer’s disease patients (Busciglio and Yankner, 1995; Reed et al., 2009; Perluigi et al., 2011; Hartley et al., 2015; Barone et al., 2017; Di Domenico et al., 2017).

With regard to oxidative stress pathways, it has been demonstrated that Down syndrome individuals have higher levels of thio-barbituric acid reactive substances, uric acid, and superoxide dismutase and catalase activity than corresponding controls (Garcez et al., 2005), and these oxidative stress parameters can trigger inflammation (Ramos-González et al., 2021), as can increased ROS production (Hussain et al., 2016). The potential of prenatal antioxidant therapy to prevent or postpone the emergence of oxidative stress disorders in the Down syndrome population is supported by the observation of early-pregnancy oxidative stress in infants with Down syndrome (Perrone et al., 2007), and also that amyloid-β deposition in Down syndrome is preceded by neuronal oxidative stress (Nunomura et al., 2000). In addition, a review study described a possible direct correlation of the role of damaged mitochondria in Down syndrome pathology with normal aging, as the onset of dementia in Down syndrome individuals resembles Alzheimer’s disease (Perluigi and Butterfield, 2012). Another study reported the potential role for the application of oxidative stress markers in the prenatal screening of T21 with the highest screening utility of plasma asprosin (Buczyńska et al., 2021). Other than measuring the levels of asprosin study under discussion also measured other oxidative stress markers in the prenatal screening of trisomy 21. The DNA/RNA oxidative stress damage products (OSDPs), advanced glycation end (AGE) products, ischemia-modified albumin (IMA), alfa-1-antitrypsin, asprosin, and vitamin D concentrations were measured in both maternal plasma and amniotic fluid in trisomy 21 (T21) and euploid pregnancies. The data of these investigations indicated increased levels of DNA/RNA oxidative stress damage products and asprosin with simultaneous decreased levels of vitamin D and A1AT in the study group. These findings support the crucial contributions of oxidative stress pathways to the pathogenesis of Down syndrome and accordingly suggest those pathways can be therapeutically targeted to attenuate associated symptoms.

To bring this all together, current review makes a narrative statement that oxidative stress is not the cause of Down syndrome, oxidative stress (OS) is demonstrably involved in complications of the Down syndrome through activation of various pathways like the AGE-RAGE and mTOR pathways and consequent inhibition of autophagy. Furthermore, in addition to being the major abnormality in the pathogenesis of Down syndrome, trisomy 21 is also involved in the upregulation of pro-oxidative stress pathways and creating an imbalance between pro-oxidants and antioxidants through excess expression of Cu/Zn-superoxide dismutase. Screening of DNA/RNA OSDPs, advanced glycation end (AGE) products, ischemia-modified albumin (IMA), alfa-1-antitrypsin, asprosin, and vitamin D concentrations in maternal plasma and amniotic fluid can play a potential role in the diagnosis of oxidative stress in pregnancy and minimizing the neurobiological changes of the fetus.

## 5 Down syndrome and neurotransmitters

**FIGURE 3:**
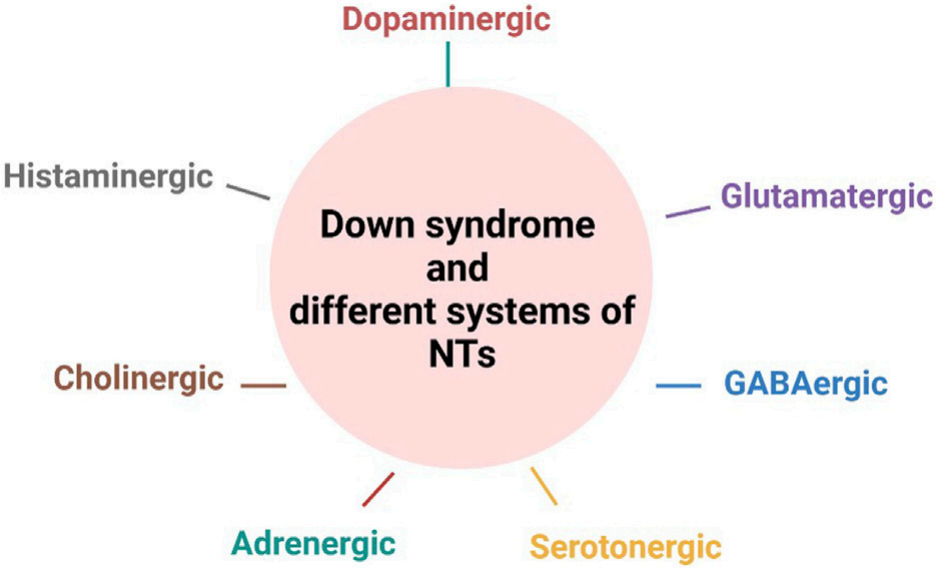
Involvement of neurotransmitter systems in the pathophysiology of Down syndrome. NT, neurotransmitters. Figure was created with BioRender.com.

A previous review (Das et al., 2014) highlighted the importance of addressing cognitive impairments in Down syndrome through the modulation of neurotransmitters. This can be done, for example, by elevating serotonin or norepinephrine levels, or by blocking the receptors of gamma-aminobutyric acid (GABA) or N-methyl-D-aspartic acid (NMDA. Here, we examine the pathological or pharmacological involvements of neurotransmitters, including dopamine and histamine, in cognition and other neurobehavioral symptoms in Down syndrome. The neurotransmitters discussed in this review include the dopamine, glutamate, GABA, serotonin, histamine adrenergic, and cholinergic systems (Figure 3).

### 5.1 Implications of the dopaminergic system in Down syndrome

**FIGURE 4:**
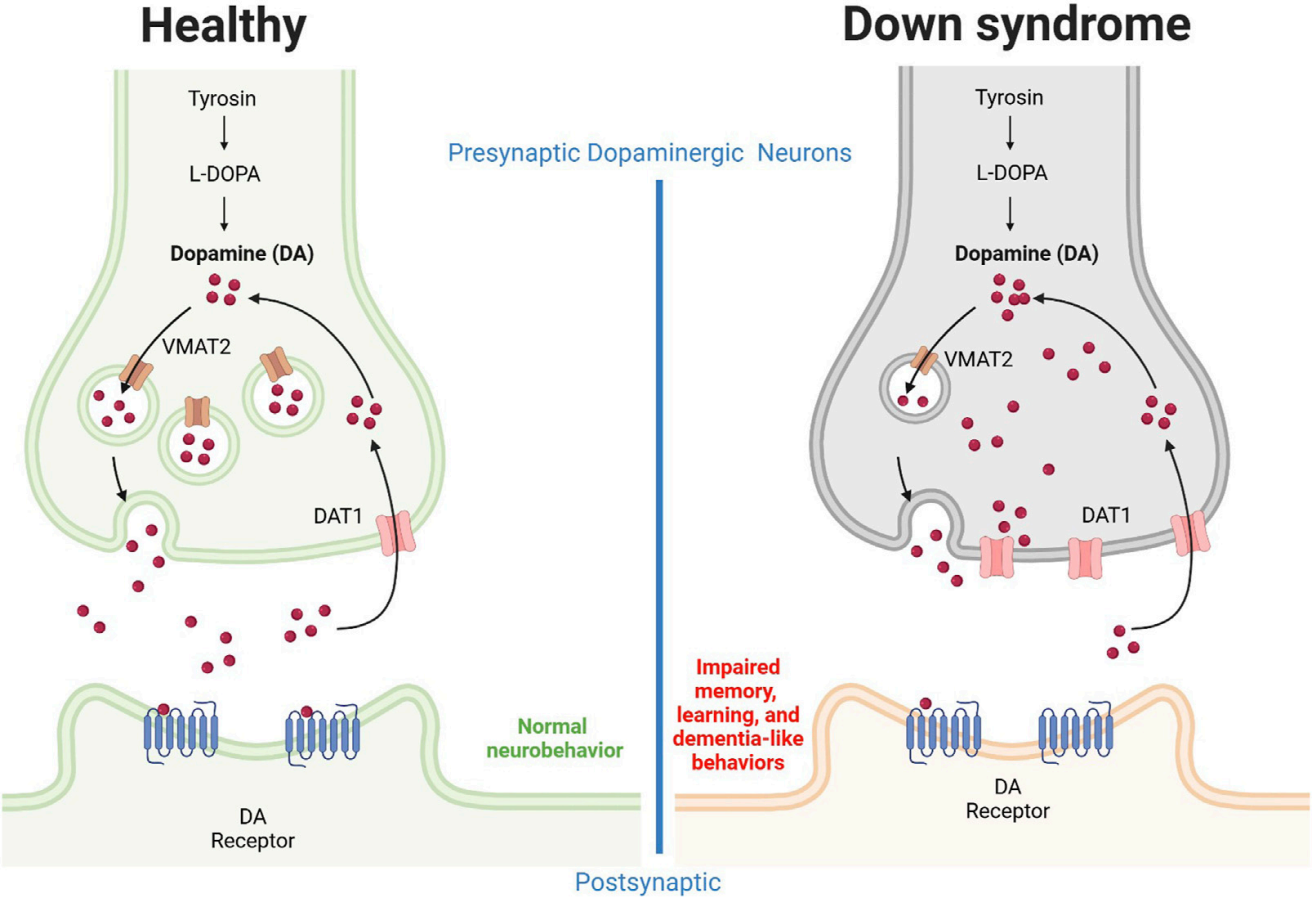
Alteration of the dopaminergic system in Down syndrome individuals. Neuronal dopamine impairment results in part from upregulation of dopamine transporter 1 and downregulation of vesicular monoamine transporter 2. The dopamine release from presynaptic neurons is decreased, possibly due to neurofibrillary degeneration and loss of pigmented dopaminergic nerve cells. In combination, these effects result in reduced extracellular dopamine concentrations, an effect associated with dysregulated memory and learning as well as dementia-like behaviors. DA, dopamine; DAT, dopamine transporter; VMAT, Vesicular monoamine transporter. Figure was created with BioRender.com.

The health outcomes of individuals with Down syndrome are influenced by dopamine neurotransmission. A marked decrease in dopamine concentration in the frontal cortex has been reported in fetal Down syndrome (Whittle et al., 2007), indicating that dysregulation of dopamine homeostasis occurs very early. A summary diagram of this dysregulation is presented in Figure 4. Concerning dopamine receptors, it has been determined that polymorphisms in dopamine receptor 4 are not linked to Down syndrome (Das Bhowmik et al., 2008), and therefore dopamine transporter 1 and vesicular monoamine transporter are more likely to be implicated in Down syndrome pathogenesis. Future pharmacological studies are warranted to explore the therapeutic effects of blocking dopamine transporter 1 on Down syndrome symptoms and associated behavioral manifestations. This strategy is expected to be associated with normalized extracellular dopamine concentrations in the brain unless the dopaminergic neurons have been damaged.

For instance, Ts1Cje model mice show enhanced flow via cerebral dopamine metabolism along with locomotor hyperactivity and sociability driven by ambient cues while in addition to this the striatum and ventral forebrain of this mouse also exhibit higher extracellular dopamine concentrations (Shimohata et al., 2017). Another study discovered the non-canonical Notch ligand Delta-like 1 to be downregulated in dopaminergic neurons, which could lead to elevated ROS levels and neuronal dopamine buildup, in part by upregulating dopamine transporter 1 (Sun et al., 2022). The authors concluded that, in individuals with Down syndrome, mitochondrial malfunction and oxidative stress may be related to dysregulated dopamine homeostasis (Sun et al., 2022). In one Down syndrome case, it was reported that dopaminergic neurons exhibited upregulation of dopamine transporter 1, downregulation of vesicular monoamine transporter 2, and defective neurite formation (Pham et al., 2018). In addition, individuals with Down syndrome show reduced dopamine release from dopaminergic neurons (Pham et al., 2018). Collectively, these findings indicate that synapse dopamine levels might be reduced in the brains of Down syndrome individuals, which in turn suggests stimulation of the dopaminergic system as a beneficial therapeutic strategy, increasing dopamine neurotransmission and extracellular concentrations and potentially ameliorating Down syndrome symptoms, including dementia. This hypothesis is further supported by the observation of neurofibrillary degeneration, loss of pigmented dopaminergic nerve cells, and significant atrophy in ten middle-aged individuals with Down syndrome (Mann et al., 1987).

### 5.2 Implications of the glutamatergic system in Down syndrome

The glutamatergic system has been implicated in Down syndrome, with potential roles of excitatory neurotransmitters in its pathogenesis. Compared to control samples, platelets and fibroblasts from individuals with Down syndrome exhibit marked decreases in glutamate uptake (Begni et al., 2003), which alteration was attributed to beta-amyloid aggregation, mitochondrial dysfunction, and amyloid precursor protein overexpression (Begni et al., 2003). Ts65Dn model mice show glutamatergic synaptic loss in the prefrontal cortex (Thomazeau et al., 2023), and a review has summarized how glutamate (specifically NDMA) receptor blockers can help attenuate memory impairment in individuals with Down syndrome (CS Costa, 2014). Likewise, in a segmental trisomy mouse model of Down syndrome, glutamate deficiency was linked to downregulation of hippocampal NMDA receptor 1 mRNA and protein expression, which in turn related to impaired neurobehavioral characteristics; in addition, the hippocampal glutamate concentration was noticeably decreased (Kaur et al., 2014). However, in fetal Down syndrome, no association was observed between frontal cortex area and altered levels of arginine, aspartate, glutamine, or glutamate within the frontal cortex (Whittle et al., 2007). Extant findings also support a role of ionotropic glutamate receptors (iGluRs) in Down syndrome-associated synaptopathy, with a detailed profile of phosphopattern changes in the glutamatergic synapse having been reported for Ts65Dn model mice (Gómez de Salazar et al., 2018). The kinase/phosphatase profiles and observed modification of iGluR phosphoresidues in Ts65Dn hippocampi suggest possible new therapeutic targets for the management of glutamatergic dysfunctions in Down syndrome (Gómez de Salazar et al., 2018).

Pre-clinical research applying memantine, an NMDA receptor blocker, yielded encouraging therapeutic results (Lockrow et al., 2011a), with long-term therapy enhancing recognition and spatial memory function abilities in Ts65Dn mice, although not to the same extent as normosomic littermate controls (Lockrow et al., 2011a). In a human study, memantine was not found to significantly enhance cognition in Down syndrome individuals as compared to controls (Islam et al., 2024); however, memantine showed promise in mice, but not in humans. Two other studies in Ts65Dn mice have reported promising results. In one, performance abnormalities on a fear conditioning test were restored by acute treatment with memantine (Costa et al., 2008), while in the other, memantine was found to pharmacologically correct the long-term synaptic depression observed in hippocampus slices (Scott-McKean and Costa, 2011). These findings provide clear support for the importance of NMDA receptors in modulating neurobiological impairments and clinical outcomes in Down syndrome; however, further investigation is still required regarding the therapeutic activity of NMDA antagonists such as memantine compared to other available treatments.

Metabotropic glutamate receptors (mGluRs) also have demonstrated alteration of expression in Down syndrome individuals. For example, elevated prenatal mGluR5 expression was observed in white matter astrocytes, which continued into the postnatal period (Iyer et al., 2014). Likewise, elevated mGluR5 expression was found in astrocytes around amyloid plaques in adult Down syndrome individuals with extensive neurodegeneration linked to Alzheimer’s disease (Iyer et al., 2014). These results reflect regulation of mGluR5 in the human hippocampal region during development, and point to an early-development role for this receptor in Down syndrome astrocytes, as well as a possible role in the pathophysiology of markers linked to Alzheimer’s disease (Iyer et al., 2014). In addition, in adult brains with Down syndrome, overabundance of immunoreactivity to the AMPA receptor subunit GluR1 has been reported (Arai et al., 1996), and mGluR5 upregulation was observed in postmortem frontal lobe tissues (Oka and Takashima, 1999). Such alterations to the glutamatergic system, along with other neurotransmitter systems, are proposed to result in synaptic modifications and cognitive abnormalities, particularly modifications relating to glutamatergic receptors (Rodríguez Ortiz et al., 2019). Taken together, these data indicate that targeting glutamatergic receptors, particularly in the hippocampus, might be a beneficial therapeutic strategy for attenuating memory and learning disabilities in individuals with Down syndrome.

### 5.3 Implications of the GABAergic system in Down syndrome

GABA receptors, as regulators of the signaling pathway, are important modulators of memory and learning functioning in individuals with Down syndrome. Interestingly, examination of the α1–3 and γ2 subunits revealed the α1, α3, and γ2 subunits to have considerably altered expression profiles in growing Down syndrome hippocampi, with the α3 subunit being most impacted; specifically, all fetal hippocampus subfields and developmental periods showed a preferential downregulation of α3 subunits, an effect linked to higher levels of amyloid precursor protein in the hippocampus (Milenkovic et al., 2018). Similarly, numerous studies in trisomic animal models of Down syndrome have demonstrated aberrant GABAergic transmission to be a major determinant of synaptic and memory impairments (Contestabile et al., 2017). One mechanism is through perturbation of GABAergic neuron genesis, an alteration that occurs during brain development and continues into maturity (Contestabile et al., 2017). Study under discussion mentioned that the failure of the CLEMATIS trial and the limited positive results from other clinical studies may be attributed to the inefficacy of the drugs (despite strong preclinical data), the limited predictive value of the Ts65Dn mouse model could also have contributed. BP27832 (Clematis) was a randomized, double-blind, placebo-controlled, multi-country phase II study to investigate the efficacy and safety of basmisanil in adults (18–30 years) and adolescents (12–17 years) with Down syndrome. Regardless of the negative outcome of the Clematis study, the insights gained on outcome measures, the feasibility of conducting international trials in Down syndrome, relationships with advocacy groups, and interactions with health authorities offer valuable information to support future clinical trials in Down syndrome and other populations with intellectual disabilities (Goeldner et al., 2022). In a rodent model of Down syndrome, long-term fluoxetine treatment in adulthood was found to correct the hippocampus synaptic plasticity and spatial memory while normalizing GABA release; hence, increased GABA release is associated with impaired cognitive properties (Begenisic et al., 2014). Changes in Gamma-Aminobutyric Acid receptor (GABAAR) subunit composition specific in humans with Down syndrome and lacking in Ts65Dn mice would lead to therapeutic failure (Bhattacharyya et al., 2009). A review article reported in 2015 explained the possible therapies to improve the intellectual disability in Down syndrome and emphaszie that sooner the initiation of the therpay better will be therapeutic outcome (Stagni et al., 2015). Another study concluded that he scientific evidence available on the pharmacological treatment of co-morbid psychiatric disorders in Down syndrome is limited to case reports and case series with very few randomized controlled trials (Palumbo and McDougle, 2018) and if pharmacological interventions are failed to treat psychiatric disorder in Down syndrome then electroconvulsive therapy should be recommended.

Correspondingly, GABA receptor antagonists have been shown to enhance cognition abilities in an animal model (Fernandez et al., 2007). Indeed, modulation of the GABAergic system, including GABAergic receptors, is strongly involved in the attenuation of dementia related to Down syndrome. For example, reported study (Braudeau et al., 2011) stated that learning and memory abilities of Ts65Dn mice can be restored by treatment with a subtype-selective inverse agonist that interacts with the benzodiazepine binding site on the GABA-A receptor. Furthermore, study under discussion explained that non-convulsant α5-selective GABA-A inverse agonists may help Down syndrome indiduals who struggle with memory and learning. Additionally, long-term treatment of Ts65Dn mice with a promnesiant GABA-A alpha5-selective inverse agonist has been demonstrated to correct the expression of immediate early genes and boost their expression during memory processing, while pharmacological targeting of the same receptor with α5IA improved memory and learning dysfunction. Furthermore, short-term treatment of the same mouse model with pentylenetetrazole, a GABA-A receptor antagonist, has shown a long-lasting pro-cognitive effect (Colas et al., 2013), while long-term treatment can correct the spatial cognition (Rueda et al., 2008). A partial inverse agonist that acts at the α5-subunit-containing GABA-A receptor is undergoing clinical trials in individuals with Down syndrome. Such receptors allow for the essential control of cognitive processes in part through efficient modulation of tonic inhibition (Rudolph and Möhler, 2014). Meanwhile, GABA receptor inhibition is associated with long-term potentiation, normalization of NMDA receptor-mediated currents, and subsequent restoration (Kleschevnikov et al., 2004). Further research remains needed concerning the interaction of the GABAergic system with other systems of neurotransmitters, such as the NMDA glutamatergic receptor, in both clinical and pre-clinical phases of Down syndrome.

### 5.4 Implications of the serotonergic system in Down syndrome

The serotonergic system exhibits alterations similar to the dopaminergic system in individuals with Down syndrome. For example, fetal Down syndrome brains showed reductions of both dopamine and serotonin in the frontal cortex region (Whittle et al., 2007). Likewise, post-mortem tissue samples from the caudate nucleus and temporal cortex of individuals with Down syndrome exhibit age-dependent serotonin reductions of 60% and 40%, respectively. These reductions were accompanied by a corresponding drop in 5-hydroxyindol-3-acetic acid, a metabolite of serotonin (Seidl et al., 1999). Furthermore, post-mortem brain samples exhibit marked elevation of serotonin transporter expression in the frontal cortex, but not the cerebellum (Gulesserian et al., 2000). It has also been reported that, in comparison to controls, people with Down syndrome have 60% less serotonin in their platelets, accompanied by a lower *Vmax* and a higher *Km* for serotonin uptake (Bayer and McCoy, 1974). Such reduction of platelet serotonin content in individuals with Down syndrome is attributed to decreased inward sodium transport (McCoy et al., 1974).

Conversely, obsessive, depressive, and apathetic behaviors in Down syndrome individuals can be treated with selective serotonin reuptake inhibitor antidepressants, with patients receiving treatment demonstrating improvements in objective metrics, including workplace productivity as reported by caregivers (Geldmacher et al., 1997). These findings are further supported by a retrospective study that found a 12-week course of selective serotonin reuptake inhibitor medication to be effective for most Down syndrome individuals, with some able to tolerate long-term use (Thom et al., 2021). It is important to note that the selective serotonin reuptake inhibitor fluoxetine reportedly restores impaired neurogenesis in the hippocampal region of the Ts65Dn mouse model (Clark et al., 2006). Additionally, when a selective serotonin uptake inhibitor was used to treat a individuals with Down syndrome, improvements in appetite and gross motor capabilities were observed (Tamasaki et al., 2016), indicating that acute regression in adolescence is not merely a general comorbidity of depression or limbic encephalitis, but rather a process directly linked to the serotonergic and cholinergic system abnormalities that are intrinsic to Down syndrome brains (Tamasaki et al., 2016). Together, extant data supports that selective serotonin uptake inhibitors might be potential therapeutic compounds for attenuating the behavioral symptoms associated with Down syndrome. Interestingly, a case report found that an increase in dietary serotonin can lower self-harming behavior in adults with Down syndrome both on the first day and after 6 months (Gedye, 1990). These findings confirm serotonin uptake proteins as having major roles in mitigating the behavioral symptoms associated with neurological disorders such as Down syndrome. Maintaining optimal serotonin levels in the synaptic clefts should be a goal when managing Down syndrome and associated behavioral manifestations.

### 5.5 Implications of the adrenergic system in Down syndrome

Norepinephrine, a partial agonist of the β1-adrenergic receptor (Conceição et al., 2021; Yuan et al., 2025), is highly implicated in the pathogenesis of Down syndrome, and numerous pre-clinical studies indicate that enhancing the norepinephrinergic system may be a viable treatment approach for Down syndrome and Alzheimer’s disease (Ponnusamy et al., 2019). For example, treatment of Ts65Dn mice with l-threo-3,4-dihydroxyphenylserine, a norepinephrine precursor that penetrates the blood-brain barrier, has been shown to recover two forms of contextual learning, fear conditioning and nest building (Salehi et al., 2009), while formoterol, a long-acting β2 adrenergic agonist, enhances cognitive performance and fosters dendritic complexity (Dang et al., 2014). The potential disease-modifying effects of norepinephrine and β-adrenergic receptor agonists in Alzheimer’s disease and Down syndrome can be explained by the reciprocal link between the inflammatory and norepinephrine-ergic systems (Ponnusamy et al., 2019). One possible connection between the central norepinephrine-ergic system and neuroinflammatory mechanisms in Alzheimer’s disease lies in the fact that both norepinephrine and the β2 adrenergic receptor agonist isoprenaline activate the β2 adrenergic receptor, which in turn promotes amyloid β peptide uptake and degradation by murine microglia (Kong et al., 2010). Therefore, norepinephrine neurotransmission might be a key player in Down syndrome pathogenesis.

Ts65Dn mice have substantial impairments in synaptic transmission of the central β-noradrenergic system, which are unique to particular regions of the brain (Dierssen et al., 1997) while central β-noradrenergic receptors in the cerebral cortex exhibit a small decrease in affinity, moreover, another study (Lockrow et al., 2011b) observed a gradual decline in the norepinephrine phenotype in locus coeruleus neurons. Subsequent research suggested that noradrenergic degeneration may contribute to the worsening memory loss, neuroinflammation, and cholinergic dysfunction experienced by individuals with Down syndrome, presenting a potential avenue for future clinical research and treatment. Norepinephrine stimulation has been suggested to prevent memory loss in Ts65Dn mice (Fortress et al., 2015), and treatment with a prodrug for norepinephrine can improve learning failure (Salehi et al., 2009). Likewise, in young-adult rats and elderly rhesus monkeys, the norepinephrine reuptake inhibitor atomoxetine enhances memory and other aspects of executive function (Callahan et al., 2019). However, little is known about the therapeutic effects of norepinephrine reuptake inhibitors on Down syndrome pathogenesis. Researchers are encouraged to test the effectiveness of norepinephrine reuptake inhibitors on neurobiological and neurobehavioral impairments associated with Down syndrome.

### 5.6 Implications of the cholinergic system in Down syndrome

The cholinergic system also has reported involvement in the pathogenesis of Down syndrome and can be pharmacologically targeted to attenuate impairments associated with Down syndrome. It has been reported that individuals with Down syndrome have a normal complement of cholinergic neurons in their brains from birth (Kish et al., 1989). Previous findings imply that memory problems in Ts65Dn mice may be caused by a reduction in the reactivity of hippocampal acetylcholine release (Chang and Gold, 2008). Remarkably some intriguing results were highlighted in a prior review, indicating that adding more choline, a precursor of acetylcholine, to the mother’s prenatal diet could be a safe and efficient method of enhancing cognitive, affective, and neural functioning in children with Down syndrome (Strupp et al., 2016). A previous study further explained that higher maternal choline intake would be beneficial for all pregnancies, this type of advice might be recommended to all pregnant women (Strupp et al., 2016). This would provide a very early intervention for those who have Down syndrome, even children born to women who are not aware they are carrying a fetus with the disorder. Notably, treatment of Ts65Dn mice with the acetylcholinesterase inhibitor physostigmine completely corrected memory deficits in the four-month-old group, but not in older animals (Chang and Gold, 2008), further supporting importance of early intervention.

Individuals with Down syndrome also exhibit elevated acetylcholinesterase activity in lymphocytes, an effect linked to a significant rise in inflammatory cytokines, including interleukins and tumor necrosis factors (Rodrigues et al., 2014). Thus, it is suggested that cross-talk between cholinergic system dysfunction and neuroinflammation might contribute to Down syndrome pathogenesis. Further evidence in favor of this theory is provided by a study on Down syndrome and dementia patients that found the median survival time for individuals using cholinesterase inhibitors to be noticeably longer than for individuals not on medication (Eady et al., 2018). It is suggested that successive evaluations of the condition status will show an early impact on preserving cognitive function.

Donepezil, an acetylcholinesterase inhibitor, has been suggested in a clinical investigation to enhance language ability in individuals with Down syndrome who do not have dementia; however, larger-scale research is required to validate this finding (Johnson et al., 2003). In children and adolescents with Down syndrome, clinical research found no difference between donepezil and a placebo (Kishnani et al., 2010), consistent with a mouse study that reported no benefit of donepezil therapy for spatial cognition (Rueda et al., 2008). Meanwhile, altered brain expression of nicotinic acetylcholine receptor (nAChR) subunits has been reported in individuals with Down syndrome, summarized in a published chapter (Engidawork et al., 2001), and agonists that target alpha 7-nAChR may have positive therapeutic benefits, as suggested in an animal model of Down syndrome (Deutsch et al., 2014). Notably, Down syndrome features increased production of the amyloidogenic Aβ1-42 peptide, which binds to α7nAChR or the lipid milieu associated with that receptor, setting off a chain of events that leads to cytotoxicity and the formation of amyloid plaques (Deutsch et al., 2015). Therefore, nAChR subunit expression and functions throughout the brain may merit research in the near future to assess their therapeutic roles in modulating neurobehavioral and neurological impairments in Down syndrome.

### 5.7 Role of the histaminergic system in Down syndrome

The histaminergic system has also been investigated in the context of Down syndrome. A reduction of histamine-releasing factor protein has been reported in the brains of individuals with Alzheimer’s disease and Down syndrome, which the authors linked to the impaired memory function in Down syndrome and Alzheimer’s disease (Kim et al., 2001). Reduced histamine in the frontal cortex of Down syndrome individuals was corroborated in a second study that associated the effect with decreased activity of histidine decarboxylase, an enzyme responsible for histamine synthesis (Schneider et al., 1997). Study under discussion further explained that both Down syndrome and Alzheimer’s disease feature similar neurobiological alterations of the histaminergic system and histaminergic deficits in the brain. In addition, histamine-N-methyl transferase is markedly reduced in the frontal cortex of Down syndrome individuals; decreasing its expression may provide a compensatory mechanism to reduce histamine degradation (Kim et al., 2002). Hence, the histaminergic system may be involved in the impairments of memory and attentional behavior observed in Down syndrome or Alzheimer’s disease. Future research should investigate the effects of increasing histamine synthesis in the brain on the neurobehavioral and molecular changes in individuals with Down syndrome.

## 6 Future therapeutic options

In addition to neurotransmitter pathways target to reduce the complexity of Down syndrome, study also reported that by altering the expression of dual-specificity tyrosine phosphorylation-regulated kinase 1A (DYRK1A) (involved in neurodevelopment) at the level of RNA, it is proposed that antisense oligonucleotides could provide a highly specific and effective treatment option addressing the intellectual disability and cognitive issues for those with Down syndrome, which further improve their overall quality of life (Murphy et al., 2024). The dose dependent inhibition of DYRK1A is an attractive target for the management of Down syndrome related neurological effects. A list of these inhibitors includes epigallocatechin gallate obtained from green tea, and harmine, which is a β-carboline alkaloid (Jarhad et al., 2018), while some synthetic DYRK1A inhibitors are summarized in published study (Liu et al., 2022). Some study emphasized the CRISPR/Cas systems and genome editing technologies play a crucial role in revolutionizing the treatment of neurodegenerative diseases, providing hope for more effective therapeutic approaches and, ultimately, better patient outcomes (Mishra et al., 2025). Recent advances in combining artificial intelligence with digital biomarkers suggest the possibility of identifying pre-symptomatic indicators of neurodegenerative diseases. Eye tracking techniques by using artificial intelligence and machine learning helped to differentiate PD patients and normal individuals (Chudzik et al., 2024). A previous study findings demonstrate that eye tracking biomarkers can reliably indicate PD motor and cognitive performance scores, offering a non-invasive method for early disease detection (Brien et al., 2023). These potential therapeutic options suggest that artificial intelligence with digital biomarkers can be utilized for the diagnosis of neurodegenerative disease.

## 7 Conclusion and future directions

Oxidative stress and inflammations are not the ultimate causes of pathogenesis in Down syndrome, as the disease stems from extra copies of genes on chromosome 21. However, oxidative stress secondary to trisomy 21, including duplication of the gene encoding Cu/Zn-superoxide dismutase, contributes to Down syndrome symptoms and complicates its prognosis through activation of pathways such as AGE-RAGE and mTOR and inhibition of autophagy. Inflammation worsens disease prognosis in a similar fashion due to increased levels of galectin-3 and HSPA 72, which activate inflammation and the immune system. Marked loss of monoaminergic neurons (dopamine, serotonin, norepinephrine, and histamine) is observed in the brains of Down syndrome animal models at pre-clinical and clinical stages. Therefore, increasing the synaptic contents of these monoamines or enhancing their neuronal biosynthesis might be beneficial therapeutic strategies by which to attenuate the dementia associated with Down syndrome. Studies also suggest inhibiting the reuptake of certain monoamines as another potential therapeutic strategy. Antagonists of glutamatergic and GABAergic receptors have also demonstrated the ability to improve cognition in Down syndrome. Finally, the cholinergic system is likewise implicated in Down syndrome pathogenesis, but the effects of its modulation need more investigation. Indeed, detailed investigation remains necessary on the role of each neurotransmitter system in the neurobiological and neurobehavioral changes observed at different age stages, including infancy, childhood, adolescence, and adulthood. Finally, the networks and interactions of proteins and signaling pathways that regulate neurotransmitters should be explored in the context of Down syndrome. This will add novel data for drug discovery and development at both clinical and pre-clinical phases. Our review article concentrates mainly only on neurotransmitter, inflammatory, and oxidative stress pathways, however, other essential mechanisms such as genetic factors, epigenetic alterations, or other molecular pathways might be involved in Down syndrome and future studies should also evaluate these mechanisms. Research on Down syndrome may still be in preclinical or early clinical phases, lack of sufficient clinical data might affect the translational therapeutic practices.

## References

[B1] Agarwal Gupta N. Kabra M. (2014). Diagnosis and management of Down syndrome. Indian J. Pediatr. 81, 560–567. 10.1007/s12098-013-1249-7 24127006

[B2] Ahmad A. Sattar M. A. Rathore H. A. Abdulla M. H. Khan S. A. Azam M. (2016). Up regulation of cystathione γ lyase and hydrogen sulphide in the myocardium inhibits the progression of isoproterenol–caffeine induced left ventricular hypertrophy in *Wistar* kyoto rats. PLoS One 11, e0150137. 10.1371/journal.pone.0150137 26963622 PMC4786159

[B3] Antonarakis S. E. Lyle R. Dermitzakis E. T. Reymond A. Deutsch S. (2004). Chromosome 21 and down syndrome: from genomics to pathophysiology. Nat. Rev. Genet. 5, 725–738. 10.1038/nrg1448 15510164

[B4] Antonarakis S. E. Skotko B. G. Rafii M. S. Strydom A. Pape S. E. Bianchi D. W. (2020). Down syndrome. Nat. Rev. Dis. Prim. 6, 9. 10.1038/s41572-019-0143-7 32029743 PMC8428796

[B5] Arai Y. Mizuguchi M. Takashima S. (1996). Excessive glutamate receptor 1 immunoreactivity in adult Down syndrome brains. Pediatr. Neurol. 15, 203–206. 10.1016/s0887-8994(96)00167-1 8916156

[B6] Arnaud P. Burdash N. Wilson G. Fudenberg H. (1976). Alpha-1-antitrypsin (Pi) types in Down’s syndrome. Clin. Genet. 10, 239–243. 10.1111/j.1399-0004.1976.tb00041.x 135662

[B7] Asim A. Kumar A. Muthuswamy S. Jain S. Agarwal S. (2015). Down syndrome: an insight of the disease. J. Biomed. Sci. 22, 41–49. 10.1186/s12929-015-0138-y 26062604 PMC4464633

[B8] Ataman A. D. Vatanoğlu-Lutz E. E. Yıldırım G. (2012). Medicine in stamps: history of Down syndrome through philately. J. Turkish Ger. Gynecol. Assoc. 13, 267–269. 10.5152/jtgga.2012.43 PMC3881714 24592054

[B9] Ayar G. Savas-Erdeve S. Azapağası E. Neşelioğlu S. Erel Ö. Çetinkaya S. (2019). Role of ischemia modified albumin serum levels as an oxidative stress marker in children with diabetic ketoacidosis. Comb. Chem. and High Throughput Screen. 22, 577–581. 10.2174/1386207322666191008214919 31595845

[B10] Barone E. Head E. Butterfield D. A. Perluigi M. (2017). HNE-modified proteins in Down syndrome: involvement in development of Alzheimer disease neuropathology. Free Radic. Biol. Med. 111, 262–269. 10.1016/j.freeradbiomed.2016.10.508 27838436 PMC5639937

[B11] Baumer N. T. O’neill M. E. (2022). Neurological and neurodevelopmental manifestations in children and adolescents with Down syndrome. Int. Rev. Res. Dev. Disabil. 63, 187–246. 10.1016/bs.irrdd.2022.09.004

[B12] Bayer S. M. Mccoy E. E. (1974). A comparison of the serotonin and ATP content in platelets from subjects with Down’s syndrome. Biochem. Med. 9, 225–232. 10.1016/0006-2944(74)90056-8 4275005

[B13] Begenisic T. Baroncelli L. Sansevero G. Milanese M. Bonifacino T. Bonanno G. (2014). Fluoxetine in adulthood normalizes GABA release and rescues hippocampal synaptic plasticity and spatial memory in a mouse model of Down syndrome. Neurobiol. Dis. 63, 12–19. 10.1016/j.nbd.2013.11.010 24269730

[B14] Begni B. Brighina L. Fumagalli L. Andreoni S. Castelli E. Francesconi C. (2003). Altered glutamate uptake in peripheral tissues from Down syndrome patients. Neurosci. Lett. 343, 73–76. 10.1016/s0304-3940(03)00260-x 12759167

[B15] Bhattacharyya A. Mcmillan E. Chen S. I. Wallace K. Svendsen C. N. (2009). A critical period in cortical interneuron neurogenesis in down syndrome revealed by human neural progenitor cells. Dev. Neurosci. 31, 497–510. 10.1159/000236899 19738365 PMC2818457

[B16] Braudeau J. Delatour B. Duchon A. Pereira P. L. Dauphinot L. De Chaumont F. (2011). Specific targeting of the GABA-A receptor α5 subtype by a selective inverse agonist restores cognitive deficits in Down syndrome mice. J. Psychopharmacol. 25, 1030–1042. 10.1177/0269881111405366 21693554 PMC3160204

[B17] Brien D. C. Riek H. C. Yep R. Huang J. Coe B. Areshenkoff C. (2023). Classification and staging of Parkinson’s disease using video-based eye tracking. Park. and Relat. Disord. 110, 105316. 10.1016/j.parkreldis.2023.105316 36822878

[B18] Buczyńska A. Sidorkiewicz I. Ławicki S. Krętowski A. J. Zbucka-Krętowska M. (2021). Prenatal screening of trisomy 21: could oxidative stress markers play a role? J. Clin. Med. 10, 2382. 10.3390/jcm10112382 34071365 PMC8198847

[B19] Busciglio J. Yankner B. A. (1995). Apoptosis and increased generation of reactive oxygen species in Down’s syndrome neurons *in vitro* . Nature 378, 776–779. 10.1038/378776a0 8524410

[B20] Butterfield D. A. Halliwell B. (2019). Oxidative stress, dysfunctional glucose metabolism and Alzheimer disease. Nat. Rev. Neurosci. 20, 148–160. 10.1038/s41583-019-0132-6 30737462 PMC9382875

[B21] Callahan P. M. Plagenhoef M. R. Blake D. T. Terry Jr A. V. (2019). Atomoxetine improves memory and other components of executive function in young-adult rats and aged rhesus monkeys. Neuropharmacology 155, 65–75. 10.1016/j.neuropharm.2019.05.016 31108108 PMC6839761

[B22] Campos C. Casado Á. (2015). Oxidative stress, thyroid dysfunction and Down syndrome. Indian J. Med. Res. 142, 113–119. 10.4103/0971-5916.164218 26354208 PMC4613432

[B23] Carvalho R. L. Boas V. F. V. Franco L. F. D. R. (2025). Effects of dual task on functional mobility in individuals with Down syndrome: a case–control study. Bull. Fac. Phys. Ther. 30, 20. 10.1186/s43161-025-00280-4

[B24] Castro-Piñero J. Carbonell-Baeza A. Martinez-Gomez D. Gómez-Martínez S. Cabanas-Sánchez V. Santiago C. (2014). Follow-up in healthy schoolchildren and in adolescents with Down syndrome: psycho-environmental and genetic determinants of physical activity and its impact on fitness, cardiovascular diseases, inflammatory biomarkers and mental health; the UP&DOWN study. BMC Public Health 14, 1–12. 10.1186/1471-2458-14-400 24761982 PMC4012062

[B25] Chang Q. Gold P. E. (2008). Age-related changes in memory and in acetylcholine functions in the hippocampus in the Ts65Dn mouse, a model of Down syndrome. Neurobiol. Learn. Mem. 89, 167–177. 10.1016/j.nlm.2007.05.007 17644430 PMC2246382

[B26] Chawla R. Goyal N. Calton R. Goyal S. (2006). Ischemia modified albumin: a novel marker for acute coronary syndrome. Indian J. Clin. Biochem. 21, 77–82. 10.1007/BF02913070 23105573 PMC3453755

[B27] Chudzik A. Śledzianowski A. Przybyszewski A. W. (2024). Machine learning and digital biomarkers can detect early stages of neurodegenerative diseases. Sensors 24, 1572. 10.3390/s24051572 38475108 PMC10934426

[B28] Clark S. Schwalbe J. Stasko M. R. Yarowsky P. J. Costa A. C. (2006). Fluoxetine rescues deficient neurogenesis in hippocampus of the Ts65Dn mouse model for Down syndrome. Exp. Neurol. 200, 256–261. 10.1016/j.expneurol.2006.02.005 16624293

[B29] Colas D. Chuluun B. Warrier D. Blank M. Wetmore D. Buckmaster P. (2013). Short-term treatment with the GABAA receptor antagonist pentylenetetrazole produces a sustained pro-cognitive benefit in a mouse model of Down’s syndrome. Br. J. Pharmacol. 169, 963–973. 10.1111/bph.12169 23489250 PMC3696321

[B30] Conceição F. Sousa D. M. Paredes J. Lamghari M. (2021). Sympathetic activity in breast cancer and metastasis: partners in crime. Bone Res. 9, 9. 10.1038/s41413-021-00137-1 33547275 PMC7864971

[B31] Contestabile A. Magara S. Cancedda L. (2017). The GABAergic hypothesis for cognitive disabilities in down syndrome. Front. Cell. Neurosci. 11, 54. 10.3389/fncel.2017.00054 28326014 PMC5339239

[B32] Coskun P. E. Busciglio J. (2012). Oxidative stress and mitochondrial dysfunction in Down’s syndrome: relevance to aging and dementia. Curr. Gerontology Geriatrics Res. 2012, 383170. 10.1155/2012/383170 PMC3350950 22611387

[B33] Costa A. Scott-Mckean J. J. Stasko M. R. (2008). Acute injections of the NMDA receptor antagonist memantine rescue performance deficits of the Ts65Dn mouse model of Down syndrome on a fear conditioning test. Neuropsychopharmacology 33, 1624–1632. 10.1038/sj.npp.1301535 17700645

[B34] Cs Costa A. (2014). The glutamatergic hypothesis for Down syndrome: the potential use of N-methyl-D-aspartate receptor antagonists to enhance cognition and decelerate neurodegeneration. CNS and Neurological Disorders-Drug Targets (Formerly Curr. Drug Targets-CNS and Neurological Disord.) 13, 16–25. 10.2174/18715273113126660183 24152324

[B35] Dang V. Medina B. Das D. Moghadam S. Martin K. J. Lin B. (2014). Formoterol, a long-acting β2 adrenergic agonist, improves cognitive function and promotes dendritic complexity in a mouse model of Down syndrome. Biol. Psychiatry 75, 179–188. 10.1016/j.biopsych.2013.05.024 23827853

[B36] Das D. Phillips C. Hsieh W. Sumanth K. Dang V. Salehi A. (2014). Neurotransmitter-based strategies for the treatment of cognitive dysfunction in Down syndrome. Prog. Neuro-Psychopharmacology Biol. Psychiatry 54, 140–148. 10.1016/j.pnpbp.2014.05.004 24842803

[B37] Das Bhowmik A. Dutta S. Sinha S. Chattopadhyay A. Mukhopadhyay K. (2008). Lack of association between Down syndrome and polymorphisms in dopamine receptor D4 and serotonin transporter genes. Neurochem. Res. 33, 1286–1291. 10.1007/s11064-007-9581-9 18270821

[B38] De Oliveira F. L. Gatto M. Bassi N. Luisetto R. Ghirardello A. Punzi L. (2015). Galectin-3 in autoimmunity and autoimmune diseases. Exp. Biol. Med. 240, 1019–1028. 10.1177/1535370215593826 PMC4935288 26142116

[B39] De Oliveira L. C. Coutinho A. M. Forlenza O. V. Buchpiguel C. A. Faria D. P. D. P. (2023). Amyloid deposition and neuroinflammation in middle-aged to older individuals with Down syndrome: a PET evaluation. Alzheimer’s and Dementia 19, e078897. 10.1002/alz.078897

[B40] Deutsch S. I. Burket J. A. Benson A. D. (2014). Targeting the α7 nicotinic acetylcholine receptor to prevent progressive dementia and improve cognition in adults with Down’s syndrome. Prog. Neuro-Psychopharmacology Biol. Psychiatry 54, 131–139. 10.1016/j.pnpbp.2014.05.011 24865150

[B41] Deutsch S. I. Burket J. A. Urbano M. R. Benson A. D. (2015). The α7 nicotinic acetylcholine receptor: a mediator of pathogenesis and therapeutic target in autism spectrum disorders and Down syndrome. Biochem. Pharmacol. 97, 363–377. 10.1016/j.bcp.2015.06.005 26074265

[B42] Di Domenico F. Barone E. Perluigi M. Butterfield D. A. (2017). The triangle of death in Alzheimer’s disease brain: the aberrant cross-talk among energy metabolism, mammalian target of rapamycin signaling, and protein homeostasis revealed by redox proteomics. Antioxidants and Redox Signal. 26, 364–387. 10.1089/ars.2016.6759 27626216

[B43] Di Domenico F. Pupo G. Mancuso C. Barone E. Paolini F. Arena A. (2015). Bach1 overexpression in Down syndrome correlates with the alteration of the HO-1/BVR-a system: insights for transition to Alzheimer’s disease. J. Alzheimer’s Dis. 44, 1107–1120. 10.3233/JAD-141254 25391381 PMC4677575

[B44] Di Domenico F. Tramutola A. Foppoli C. Head E. Perluigi M. Butterfield D. A. (2018). mTOR in Down syndrome: role in Aß and tau neuropathology and transition to Alzheimer disease-like dementia. Free Radic. Biol. Med. 114, 94–101. 10.1016/j.freeradbiomed.2017.08.009 28807816 PMC5748251

[B45] Dierssen M. Fructuoso M. Martínez De Lagrán M. Perluigi M. Barone E. (2020). Down syndrome is a metabolic disease: altered insulin signaling mediates peripheral and brain dysfunctions. Front. Neurosci. 14, 670. 10.3389/fnins.2020.00670 32733190 PMC7360727

[B46] Dierssen M. Vallina I. F. Baamonde C. García-Calatayud S. Lumbreras M. A. Flórez J. (1997). Alterations of central noradrenergic transmission in Ts65Dn mouse, a model for Down syndrome. Brain Res. 749, 238–244. 10.1016/s0006-8993(96)01173-0 9138724

[B47] Dursun A. Okumus N. Zenciroglu A. (2012). Ischemia-modified albumin (IMA): could it be useful to predict perinatal asphyxia? J. Maternal-Fetal and Neonatal Med. 25, 2401–2405. 10.3109/14767058.2012.697943 22642562

[B48] Eady N. Sheehan R. Rantell K. Sinai A. Bernal J. Bohnen I. (2018). Impact of cholinesterase inhibitors or memantine on survival in adults with Down syndrome and dementia: clinical cohort study. Br. J. Psychiatry 212, 155–160. 10.1192/bjp.2017.21 29486820

[B49] Ekundayo B. E. Obafemi T. O. Adewale O. B. Obafemi B. A. Oyinloye B. E. Ekundayo S. K. (2024). Oxidative stress, endoplasmic reticulum stress and apoptosis in the pathology of Alzheimer’s disease. Cell Biochem. Biophysics 82, 457–477. 10.1007/s12013-024-01248-2 38472715

[B50] Engidawork E. Gulesserian T. Balic N. Cairns N. Lubec G. (2001). Changes in nicotinic acetylcholine receptor subunits expression in brain of patients with Down syndrome and Alzheimer’s disease. Vienna: Springer. 10.1007/978-3-7091-6262-0_17 11771745

[B51] Fernandez F. Morishita W. Zuniga E. Nguyen J. Blank M. Malenka R. C. (2007). Pharmacotherapy for cognitive impairment in a mouse model of Down syndrome. Nat. Neurosci. 10, 411–413. 10.1038/nn1860 17322876

[B52] Flores-Aguilar L. Iulita M. F. Kovecses O. Torres M. D. Levi S. M. Zhang Y. (2020). Evolution of neuroinflammation across the lifespan of individuals with Down syndrome. Brain 143, 3653–3671. 10.1093/brain/awaa326 33206953 PMC7805813

[B53] Fortress A. M. Hamlett E. D. Vazey E. M. Aston-Jones G. Cass W. A. Boger H. A. (2015). Designer receptors enhance memory in a mouse model of Down syndrome. J. Neurosci. 35, 1343–1353. 10.1523/JNEUROSCI.2658-14.2015 25632113 PMC4308587

[B54] Fructuoso M. Rachdi L. Philippe E. Denis R. Magnan C. Le Stunff H. (2018). Increased levels of inflammatory plasma markers and obesity risk in a mouse model of Down syndrome. Free Radic. Biol. Med. 114, 122–130. 10.1016/j.freeradbiomed.2017.09.021 28958596

[B55] Garcez M. E. Peres W. Salvador M. (2005). “Oxidative stress and hematologic and biochemical parameters in individuals with Down syndrome,” in Mayo clinic proceedings (Elsevier), 1607–1611. 10.4065/80.12.1607 16342654

[B56] Gardiner K. J. (2010). Molecular basis of pharmacotherapies for cognition in Down syndrome. Trends Pharmacol. Sci. 31, 66–73. 10.1016/j.tips.2009.10.010 19963286 PMC2815198

[B57] Garlet T. R. Parisotto E. B. De Medeiros G. D. S. Pereira L. C. R. Dalmarco E. M. Dalmarco J. B. (2013). Systemic oxidative stress in children and teenagers with Down syndrome. Life Sci. 93, 558–563. 10.1016/j.lfs.2013.08.017 24004546

[B58] Gedye A. (1990). Dietary increase in serotonin reduces self-injurious behaviour in a Down’s syndrome adult. J. Intellect. Disabil. Res. 34, 195–203. 10.1111/j.1365-2788.1990.tb01529.x 2140418

[B59] Geldmacher D. S. Lerner A. J. Voci J. M. Noelker E. A. Somple L. C. Whitehouse P. J. (1997). Treatment of functional decline in adults with Down syndrome using selective serotonin-reuptake inhibitor drugs. J. Geriatric Psychiatry Neurology 10, 99–104. 10.1177/089198879701000302 9322131

[B60] Ghaffarpour M. Karami-Zarandi M. Rahdar H. A. Feyisa S. G. Taki E. (2024). Periodontal disease in down syndrome: predisposing factors and potential non-surgical therapeutic approaches. J. Clin. Laboratory Analysis 38, e25002. 10.1002/jcla.25002 PMC10829694 38254289

[B61] Goeldner C. Kishnani P. S. Skotko B. G. Casero J. L. Hipp J. F. Derks M. (2022). A randomized, double-blind, placebo-controlled phase II trial to explore the effects of a GABAA-α5 NAM (basmisanil) on intellectual disability associated with Down syndrome. J. Neurodev. Disord. 14, 10. 10.1186/s11689-022-09418-0 35123401 PMC8903644

[B62] Gómez De Salazar M. Grau C. Ciruela F. Altafaj X. (2018). Phosphoproteomic alterations of ionotropic glutamate receptors in the hippocampus of the Ts65Dn mouse model of Down syndrome. Front. Mol. Neurosci. 11, 226. 10.3389/fnmol.2018.00226 30140203 PMC6095006

[B63] Gulesserian T. Engidawork E. Cairns N. Lubec G. (2000). Increased protein levels of serotonin transporter in frontal cortex of patients with Down syndrome. Neurosci. Lett. 296, 53–57. 10.1016/s0304-3940(00)01624-4 11099832

[B64] Hajjo R. Sabbah D. A. Abusara O. H. Al Bawab A. Q. (2022). A review of the recent advances in Alzheimer’s disease research and the utilization of network biology approaches for prioritizing diagnostics and therapeutics. Diagnostics 12, 2975. 10.3390/diagnostics12122975 36552984 PMC9777434

[B65] Halliwell B. Gutteridge J. M. (2015). Free radicals in biology and medicine. United States: Oxford University Press.

[B66] Hamlett E. D. Hjorth E. Ledreux A. Gilmore A. Schultzberg M. Granholm A. C. (2020). RvE1 treatment prevents memory loss and neuroinflammation in the Ts65Dn mouse model of Down syndrome. Glia 68, 1347–1360. 10.1002/glia.23779 31944407 PMC7205572

[B67] Hamlett E. D. Ledreux A. Potter H. Chial H. J. Patterson D. Espinosa J. M. (2018). Exosomal biomarkers in Down syndrome and Alzheimer’s disease. Free Radic. Biol. Med. 114, 110–121. 10.1016/j.freeradbiomed.2017.08.028 28882786 PMC6135098

[B68] Hartley D. Blumenthal T. Carrillo M. Dipaolo G. Esralew L. Gardiner K. (2015). Down syndrome and Alzheimer’s disease: common pathways, common goals. Alzheimer’s and Dementia 11, 700–709. 10.1016/j.jalz.2014.10.007 PMC4817997 25510383

[B69] Hussain T. Tan B. Yin Y. Blachier F. Tossou M. C. Rahu N. (2016). Oxidative stress and inflammation: what polyphenols can do for us? Oxidative Med. Cell. Longev. 2016, 7432797. 10.1155/2016/7432797 PMC5055983 27738491

[B70] Ishihara K. Kanai S. Sago H. Yamakawa K. Akiba S. (2014). Comparative proteomic profiling reveals aberrant cell proliferation in the brain of embryonic Ts1Cje, a mouse model of Down syndrome. Neuroscience 281, 1–15. 10.1016/j.neuroscience.2014.09.039 25261685

[B71] Islam Z. Ghazanfar S. Gangat S. A. Marfani W. B. Al'saani S. a.J. Rahmat Z. S. (2024). The role of Memantine in slowing cognitive decline in patients with Down syndrome–A systematic review and meta analysis. Brain Disord. 13, 100114. 10.1016/j.dscb.2023.100114

[B72] Iyer A. Van Scheppingen J. Milenkovic I. Anink J. Lim D. Genazzani A. (2014). Metabotropic glutamate receptor 5 in Down’s syndrome hippocampus during development: increased expression in astrocytes. Curr. Alzheimer Res. 11, 694–705. 10.2174/1567205011666140812115423 25115540

[B73] Izzo A. Mollo N. Nitti M. Paladino S. Calì G. Genesio R. (2018). Mitochondrial dysfunction in Down syndrome: molecular mechanisms and therapeutic targets. Mol. Med. 24, 2–8. 10.1186/s10020-018-0004-y 30134785 PMC6016872

[B74] Jarhad D. B. Mashelkar K. K. Kim H.-R. Noh M. Jeong L. S. (2018). Dual-specificity tyrosine phosphorylation-regulated kinase 1A (DYRK1A) inhibitors as potential therapeutics. J. Med. Chem. 61, 9791–9810. 10.1021/acs.jmedchem.8b00185 29985601

[B75] Johnson N. Fahey C. Chicoine B. Chong G. Gitelman D. (2003). Effects of donepezil on cognitive functioning in Down syndrome. Am. J. Ment. Retard. 108, 367–372. 10.1352/0895-8017(2003)108<367:EODOCF>2.0.CO;2 14561111

[B76] Kaur G. Sharma A. Xu W. Gerum S. Alldred M. J. Subbanna S. (2014). Glutamatergic transmission aberration: a major cause of behavioral deficits in a murine model of Down’s syndrome. J. Neurosci. 34, 5099–5106. 10.1523/JNEUROSCI.5338-13.2014 24719089 PMC3983795

[B77] Kazemi M. Salehi M. Kheirollahi M. (2016). Down syndrome: current status, challenges and future perspectives. Int. J. Mol. Cell. Med. 5, 125–133. 27942498 PMC5125364

[B78] Kim S. H. Cairns N. Fountoulakisc M. Lubec G. (2001). Decreased brain histamine-releasing factor protein in patients with Down syndrome and Alzheimer’s disease. Neurosci. Lett. 300, 41–44. 10.1016/s0304-3940(01)01545-2 11172935

[B79] Kim S. H. Krapfenbauer K. Cheon M. S. Fountoulakis M. Cairns N. J. Lubec G. (2002). Human brain cytosolic histamine-N-methyltransferase is decreased in Down syndrome and increased in Pick’s disease. Neurosci. Lett. 321, 169–172. 10.1016/s0304-3940(02)00051-4 11880199

[B80] Kish S. Karlinsky H. Becker L. Gilbert J. Rebbetoy M. Chang L. J. (1989). Down’s syndrome individuals begin life with normal levels of brain cholinergic markers. J. Neurochem. 52, 1183–1187. 10.1111/j.1471-4159.1989.tb01864.x 2522539

[B81] Kishnani P. S. Heller J. H. Spiridigliozzi G. A. Lott I. Escobar L. Richardson S. (2010). Donepezil for treatment of cognitive dysfunction in children with Down syndrome aged 10–17. Am. J. Med. Genet. Part A 152, 3028–3035. 10.1002/ajmg.a.33730 21108390

[B82] Kleschevnikov A. M. Belichenko P. V. Villar A. J. Epstein C. J. Malenka R. C. Mobley W. C. (2004). Hippocampal long-term potentiation suppressed by increased inhibition in the Ts65Dn mouse, a genetic model of Down syndrome. J. Neurosci. 24, 8153–8160. 10.1523/JNEUROSCI.1766-04.2004 15371516 PMC6729789

[B83] Kong Y. Ruan L. Qian L. Liu X. Le Y. (2010). Norepinephrine promotes microglia to uptake and degrade amyloid β peptide through upregulation of mouse formyl peptide receptor 2 and induction of insulin-degrading enzyme. J. Neurosci. 30, 11848–11857. 10.1523/JNEUROSCI.2985-10.2010 20810904 PMC6633413

[B84] Landreth G. Jiang Q. Mandrekar S. Heneka M. (2008). PPARgamma agonists as therapeutics for the treatment of Alzheimer’s disease. Neurotherapeutics 5, 481–489. 10.1016/j.nurt.2008.05.003 18625459 PMC2593876

[B85] Li J.-G. Leone A. Servili M. Praticò D. (2024). Extra virgin olive oil beneficial effects on memory, synaptic function, and neuroinflammation in a mouse model of Down syndrome. J. Alzheimer’s Dis. 102, 35–43. 10.1177/13872877241283675 39497304

[B86] Liu T. Wang Y. Wang J. Ren C. Chen H. Zhang J. (2022). DYRK1A inhibitors for disease therapy: current status and perspectives. Eur. J. Med. Chem. 229, 114062. 10.1016/j.ejmech.2021.114062 34954592

[B87] Lockrow J. Boger H. Bimonte-Nelson H. Granholm A.-C. (2011a). Effects of long-term memantine on memory and neuropathology in Ts65Dn mice, a model for Down syndrome. Behav. Brain Res. 221, 610–622. 10.1016/j.bbr.2010.03.036 20363261 PMC2928411

[B88] Lockrow J. Boger H. Gerhardt G. Aston-Jones G. Bachman D. Granholm A.-C. (2011b). A noradrenergic lesion exacerbates neurodegeneration in a Down syndrome mouse model. J. Alzheimer’s Dis. 23, 471–489. 10.3233/JAD-2010-101218 21098982 PMC3991557

[B89] Malak R. Kostiukow A. Krawczyk-Wasielewska A. Mojs E. Samborski W. (2015). Delays in motor development in children with Down syndrome. Med. Sci. Monit. Int. Med. J. Exp. Clin. Res. 21, 1904–1910. 10.12659/MSM.893377 PMC4500597 26132100

[B90] Malle L. Patel R. S. Martin-Fernandez M. Stewart O. J. Philippot Q. Buta S. (2023). Autoimmunity in Down’s syndrome via cytokines, CD4 T cells and CD11c+ B cells. Nature 615, 305–314. 10.1038/s41586-023-05736-y 36813963 PMC9945839

[B91] Mann D. Yates P. Marcyniuk B. (1987). Dopaminergic neurotransmitter systems in Alzheimer’s disease and in Down’s syndrome at middle age. J. Neurology, Neurosurg. and Psychiatry 50, 341–344. 10.1136/jnnp.50.3.341 PMC1031800 2951499

[B92] Manna C. Officioso A. Trojsi F. Tedeschi G. Leoncini S. Signorini C. (2016). Increased non-protein bound iron in Down syndrome: contribution to lipid peroxidation and cognitive decline. Free Radic. Res. 50, 1422–1431. 10.1080/10715762.2016.1253833 27785947

[B93] Marderstein A. R. De Zuani M. Moeller R. Bezney J. Padhi E. M. Wong S. (2024). Single-cell multi-omics map of human fetal blood in Down syndrome. Nature 634, 104–112. 10.1038/s41586-024-07946-4 39322663 PMC11446839

[B94] Mccoy E. E. Segal D. J. Bayer S. M. Strynadka K. D. (1974). Decreased ATPase and increased sodium content of platelets in Down’s syndrome: relation to decreased serotonin content. N. Engl. J. Med. 291, 950–953. 10.1056/NEJM197410312911807 4278048

[B95] Milenkovic I. Stojanovic T. Aronica E. Fülöp L. Bozso Z. Máté Z. (2018). GABA A receptor subunit deregulation in the hippocampus of human foetuses with Down syndrome. Brain Struct. Funct. 223, 1501–1518. 10.1007/s00429-017-1563-3 29168008 PMC5869939

[B96] Mishra S. K. Chang H.-M. Obaid A. A. Singh S. K. (2025). “Advances in CRISPER/Cas system and genome editing technologies for the treatment of neurodegenerative diseases,” in Genome editing for neurodegenerative diseases (Elsevier), 69–90.

[B97] Muchová J. Zitnanova I. Durackova Z. (2014). Oxidative stress and Down syndrome. Do antioxidants play a role in therapy? Physiological Res. 63, 535–542. 10.33549/physiolres.932722 24908086

[B98] Murphy A. J. Wilton S. D. Aung-Htut M. T. Mcintosh C. S. (2024). Down syndrome and DYRK1A overexpression: relationships and future therapeutic directions. Front. Mol. Neurosci. 17, 1391564. 10.3389/fnmol.2024.1391564 39114642 PMC11303307

[B99] Nunomura A. Perry G. Pappolla M. A. Friedland R. P. Hirai K. Chiba S. (2000). Neuronal oxidative stress precedes amyloid-β deposition in Down syndrome. J. Neuropathology and Exp. Neurology 59, 1011–1017. 10.1093/jnen/59.11.1011 11089579

[B100] Oka A. Takashima S. (1999). The up-regulation of metabotropic glutamate receptor 5 (mGluR5) in Down’s syndrome brains. Acta Neuropathol. 97, 275–278. 10.1007/s004010050985 10090675

[B101] Pallardó F. V. Degan P. D’ischia M. Kelly F. J. Zatterale A. Calzone R. (2006). Multiple evidence for an early age pro-oxidant state in Down Syndrome patients. Biogerontology 7, 211–220. 10.1007/s10522-006-9002-5 16612664

[B102] Palumbo M. L. Mcdougle C. J. (2018). Pharmacotherapy of Down syndrome. Expert Opin. Pharmacother. 19, 1875–1889. 10.1080/14656566.2018.1529167 30257591

[B103] Perluigi M. Butterfield D. A. (2012). Oxidative stress and Down syndrome: a route toward Alzheimer-like dementia. Curr. Gerontology Geriatrics Res. 2012, 724904. 10.1155/2012/724904 PMC3235450 22203843

[B104] Perluigi M. Di Domenico F. Fiorini A. Cocciolo A. Giorgi A. Foppoli C. (2011). Oxidative stress occurs early in Down syndrome pregnancy: a redox proteomics analysis of amniotic fluid. Proteomics–Clinical Appl. 5, 167–178. 10.1002/prca.201000121 21360684

[B105] Perluigi M. Pupo G. Tramutola A. Cini C. Coccia R. Barone E. (2014). Neuropathological role of PI3K/Akt/mTOR axis in Down syndrome brain. Biochimica Biophysica Acta (BBA)-Molecular Basis Dis. 1842, 1144–1153. 10.1016/j.bbadis.2014.04.007 PMC4062876 24735980

[B106] Perrone S. Longini M. Bellieni C. Centini G. Kenanidis A. De Marco L. (2007). Early oxidative stress in amniotic fluid of pregnancies with Down syndrome. Clin. Biochem. 40, 177–180. 10.1016/j.clinbiochem.2006.10.019 17208212

[B107] Pham T. T. M. Kato H. Yamaza H. Masuda K. Hirofuji Y. Sato H. (2018). Altered development of dopaminergic neurons differentiated from stem cells from human exfoliated deciduous teeth of a patient with Down syndrome. BMC Neurol. 18, 132–139. 10.1186/s12883-018-1140-2 30170556 PMC6117917

[B108] Ponnusamy R. Mcnerney M. W. Moghadam S. Salehi A. (2019). Assessing disease-modifying effects of norepinephrine in Down syndrome and Alzheimer’s disease. Brain Res. 1702, 3–11. 10.1016/j.brainres.2017.09.035 29102776

[B109] Rajasegaran S. Ahmad N. A. Tan S. K. Lechmiannandan A. Tan Y.-W. Sanmugam A. (2024). A multi-center cross-sectional comparison of parent-reported quality of life and bowel function between anorectal malformation and Hirschsprung’s disease patients with versus those without Down syndrome. Pediatr. Surg. Int. 40, 209. 10.1007/s00383-024-05792-z 39046543

[B110] Ram G. Chinen J. (2011). Infections and immunodeficiency in Down syndrome. Clin. and Exp. Immunol. 164, 9–16. 10.1111/j.1365-2249.2011.04335.x 21352207 PMC3074212

[B111] Ramos-González E. Bitzer-Quintero O. Ortiz G. Hernández-Cruz J. Ramírez-Jirano L. (2021). Relationship between inflammation and oxidative stress and its effect on multiple sclerosis. Neurología. 39 (3), 292–301. 10.1016/j.nrleng.2021.10.010 38553104

[B112] Rastogi M. (2021). Multi-omics approach in Down syndrome resolves new regulators in two regions of the human brain. Università degli studi di Genova.

[B113] Reed T. T. Pierce W. M. Jr Turner D. M. Markesbery W. R. Allan Butterfield D. (2009). Proteomic identification of nitrated brain proteins in early Alzheimer’s disease inferior parietal lobule. J. Cell. Mol. Med. 13, 2019–2029. 10.1111/j.1582-4934.2008.00478.x 18752637 PMC2819643

[B114] Rodrigues R. Debom G. Soares F. Machado C. Pureza J. Peres W. (2014). Alterations of ectonucleotidases and acetylcholinesterase activities in lymphocytes of Down syndrome subjects: relation with inflammatory parameters. Clin. Chim. acta 433, 105–110. 10.1016/j.cca.2014.03.002 24631131

[B115] Rodríguez-Baz Í. Benejam B. Morcillo-Nieto A. O. Vaqué-Alcázar L. Arriola-Infante J. E. Camacho V. (2025). Posterior cortical atrophy due to Alzheimer disease in a person with down syndrome: a case report. Neurology 104, e210179. 10.1212/wnl.0000000000210179 39689339

[B116] Rodríguez Ortiz A. R. Saldarriaga M. a.B. Villegas J. C. M. García-Vallejo F. (2019). Temporal and spatial differential expression of glutamate receptor genes in the brain of down syndrome. Gene Regul. 10.5772/intechopen.82446

[B117] Roizen N. J. Patterson D. (2003). Down’s syndrome. Lancet 361, 1281–1289. 10.1016/S0140-6736(03)12987-X 12699967

[B118] Rudolph U. Möhler H. (2014). GABAA receptor subtypes: therapeutic potential in Down syndrome, affective disorders, schizophrenia, and autism. Annu. Rev. Pharmacol. Toxicol. 54, 483–507. 10.1146/annurev-pharmtox-011613-135947 24160694 PMC3997216

[B119] Rueda N. Flórez J. Martinez-Cue C. (2008). Chronic pentylenetetrazole but not donepezil treatment rescues spatial cognition in Ts65Dn mice, a model for Down syndrome. Neurosci. Lett. 433, 22–27. 10.1016/j.neulet.2007.12.039 18226451

[B120] Salehi A. Faizi M. Colas D. Valletta J. Laguna J. Takimoto-Kimura R. (2009). Restoration of norepinephrine-modulated contextual memory in a mouse model of Down syndrome. Sci. Transl. Med. 1, 7ra17–17ra17. 10.1126/scitranslmed.3000258 20368182

[B121] Schneider C. Risser D. Kirchner L. Kitzmüller E. Cairns N. Prast H. (1997). Similar deficits of central histaminergic system in patients with Down syndrome and Alzheimer disease. Neurosci. Lett. 222, 183–186. 10.1016/s0304-3940(97)13379-1 9148245

[B122] Scott-Mckean J. J. Costa A. C. (2011). Exaggerated NMDA mediated LTD in a mouse model of Down syndrome and pharmacological rescuing by memantine. Learn. and Mem. 18, 774–778. 10.1101/lm.024182.111 PMC3222894 22101180

[B123] Seidl R. Kaehler S. Prast H. Singewald N. Cairns N. Gratzer M. (1999). Serotonin (5-HT) in brains of adult patients with Down syndrome. Vienna: Springer. 10.1007/978-3-7091-6380-1_14 10666678

[B124] Shimohata A. Ishihara K. Hattori S. Miyamoto H. Morishita H. Ornthanalai G. (2017). Ts1Cje Down syndrome model mice exhibit environmental stimuli-triggered locomotor hyperactivity and sociability concurrent with increased flux through central dopamine and serotonin metabolism. Exp. Neurol. 293, 1–12. 10.1016/j.expneurol.2017.03.009 28336394

[B125] Sinet P. Theophile D. Rahmani Z. Chettouh Z. Blouin J. Prieur M. (1994). Mapping of the Down syndrome phenotype on chromosome 21 at the molecular level. Biomed. and Pharmacother. 48, 247–252. 10.1016/0753-3322(94)90140-6 7999986

[B126] Skotko B. G. Kishnani P. S. Capone G. T. Group D. S. D. S. (2009). Prenatal diagnosis of Down syndrome: how best to deliver the news. Am. J. Med. Genet. Part A 149, 2361–2367. 10.1002/ajmg.a.33082 19787699

[B127] Stagni F. Giacomini A. Guidi S. Ciani E. Bartesaghi R. (2015). Timing of therapies for Down syndrome: the sooner, the better. Front. Behav. Neurosci. 9, 265. 10.3389/fnbeh.2015.00265 26500515 PMC4594009

[B128] Strupp B. J. Powers B. E. Velazquez R. Ash J. A. Kelley C. M. Alldred M. J. (2016). Maternal choline supplementation: a potential prenatal treatment for Down syndrome and Alzheimer’s disease. Curr. Alzheimer Res. 13, 97–106. 10.2174/1567205012666150921100311 26391046 PMC4733524

[B129] Strydom A. Dickinson M. J. Shende S. Pratico D. Walker Z. (2009). Oxidative stress and cognitive ability in adults with Down syndrome. Prog. Neuro-Psychopharmacology Biol. Psychiatry 33, 76–80. 10.1016/j.pnpbp.2008.10.006 18983885

[B130] Sun X. Kato H. Sato H. Han X. Hirofuji Y. Kato T. A. (2022). Dopamine-related oxidative stress and mitochondrial dysfunction in dopaminergic neurons differentiated from deciduous teeth-derived stem cells of children with Down syndrome. FASEB BioAdvances 4, 454–467. 10.1096/fba.2021-00086 35812076 PMC9254221

[B131] Tamasaki A. Saito Y. Ueda R. Ohno K. Yokoyama K. Satake T. (2016). Effects of donepezil and serotonin reuptake inhibitor on acute regression during adolescence in Down syndrome. Brain Dev. 38, 113–117. 10.1016/j.braindev.2015.06.006 26143664

[B132] Tarani L. Carito V. Ferraguti G. Petrella C. Greco A. Ralli M. (2020). Neuroinflammatory markers in the serum of prepubertal children with Down syndrome. J. Immunol. Res. 2020, 6937154. 10.1155/2020/6937154 32280719 PMC7125499

[B133] Thom R. P. Palumbo M. L. Thompson C. Mcdougle C. J. Ravichandran C. T. (2021). Selective serotonin reuptake inhibitors for the treatment of depression in adults with Down syndrome: a preliminary retrospective chart review study. Brain Sci. 11, 1216. 10.3390/brainsci11091216 34573236 PMC8469816

[B134] Thomazeau A. Lassalle O. Manzoni O. J. (2023). Glutamatergic synaptic deficits in the prefrontal cortex of the Ts65Dn mouse model for Down syndrome. Front. Neurosci. 17, 1171797. 10.3389/fnins.2023.1171797 37841687 PMC10569174

[B135] Thomson P. (2007). “Down will come baby”: prenatal stress, primitive defenses and gestational dysregulation. J. Trauma and Dissociation 8, 85–113. 10.1300/J229v08n03_05 18032345

[B136] Veloso K. M. Mouchrek M. M. De Sousa J. A. Ribeiro C. C. Rodrigues V. P. Benatti B. B. (2025). Association between serum levels of inflammatory mediators and periodontitis severity in people with Down syndrome. Cytokine 189, 156910. 10.1016/j.cyto.2025.156910 40054018

[B137] Wang T. Xu J. Wang L. Cui X. Yan Y. Tang Q. (2022). Prenatal diagnosis: the main advances in the application of identification of biomarkers based on multi-omics. IntechOpen. 10.5772/intechopen.104981

[B138] Whittle N. Sartori S. B. Dierssen M. Lubec G. Singewald N. (2007). Fetal Down syndrome brains exhibit aberrant levels of neurotransmitters critical for normal brain development. Pediatrics 120, e1465–e1471. 10.1542/peds.2006-3448 17998315

[B139] Wilcock D. M. (2012). Neuroinflammation in the aging down syndrome brain; lessons from Alzheimer’ s disease. Curr. gerontology geriatrics Res. 2012, 170276. 10.1155/2012/170276 PMC3290800 22454637

[B140] Wilcock D. M. Griffin W. S. T. (2013). Down’s syndrome, neuroinflammation, and Alzheimer neuropathogenesis. J. Neuroinflammation 10, 84–10. 10.1186/1742-2094-10-84 23866266 PMC3750399

[B141] Wilcock D. M. Hurban J. Helman A. M. Sudduth T. L. Mccarty K. L. Beckett T. L. (2015). Down syndrome individuals with Alzheimer’s disease have a distinct neuroinflammatory phenotype compared to sporadic Alzheimer’s disease. Neurobiol. Aging 36, 2468–2474. 10.1016/j.neurobiolaging.2015.05.016 26103884 PMC4602365

[B142] Wiseman F. K. Pulford L. J. Barkus C. Liao F. Portelius E. Webb R. (2018). Trisomy of human chromosome 21 enhances amyloid-β deposition independently of an extra copy of APP. Brain 141, 2457–2474. 10.1093/brain/awy159 29945247 PMC6061702

[B143] Yahia S. El-Farahaty R. M. El-Hawary A. K. El-Hussiny M. A. Abdel-Maseih H. El-Dahtory F. (2012). Leptin, insulin and thyroid hormones in a cohort of Egyptian obese Down syndrome children: a comparative study. BMC Endocr. Disord. 12, 22–27. 10.1186/1472-6823-12-22 23067442 PMC3528445

[B144] Yuan X. Li Y. Cong L. Yang L. Zhang Y. Zhang Z. (2025). Norepinephrine regulates epithelial-derived neurotrophins expression and sensory nerve regeneration through ADRB2 receptor. Commun. Biol. 8, 481. 10.1038/s42003-025-07903-5 40121310 PMC11929770

